# Alcohol Motor Blocks: Case Series and a Narrative Review

**DOI:** 10.7759/cureus.21575

**Published:** 2022-01-24

**Authors:** Moses Koh, Tan Y Leng

**Affiliations:** 1 Rehabilitation Medicine/General Medicine, Sengkang General Hospital, Singapore, SGP; 2 Rehabilitation Medicine, Singapore General Hospital, Singapore, SGP

**Keywords:** stroke, alcohol motor block, alcohol neurolysis, contractures, spasticity

## Abstract

Alcohol neurolysis and intramuscular blocks are interventions for spasticity management. Here, we illustrate two clinical cases with spasticity impeding ease of care and pain which required selective alcohol intramuscular blocks with alcohol neurolysis. Post-interventions, both cases demonstrated improvement in pain and joint range of motion which facilitated better positioning and reduced caregiver burden. Pertinent learning points from alcohol neurolysis with intramuscular blocks are discussed concerning therapeutic effectiveness and intervention safety.

## Introduction

Alcohol plays an important role in neurolysis and intramuscular blocks for the treatment of spasticity [1,2]. Neurolysis using alcohol or phenol has been extensively described in neurorehabilitation; however, intramuscular blocks using these pharmacological injectates have been relatively less discussed. Focal alcohol intramuscular treatment improves spasticity and function in a wide spectrum of conditions ranging from stroke to spinal cord injuries [2,3]. Here, we highlight two clinical cases that demonstrated improved spasticity, joint range of motion (ROM), and pain from focal intramuscular blocks with alcohol guided by nerve stimulator and ultrasound. The first case involved the upper extremities while the latter described lower extremity blocks. In both patients, after alcohol treatment, the clinical improvements were sustained at six weeks and three months. Moreover, the respective caregivers reported easier nursing care.

## Case presentation

Case one

**Figure 1:**
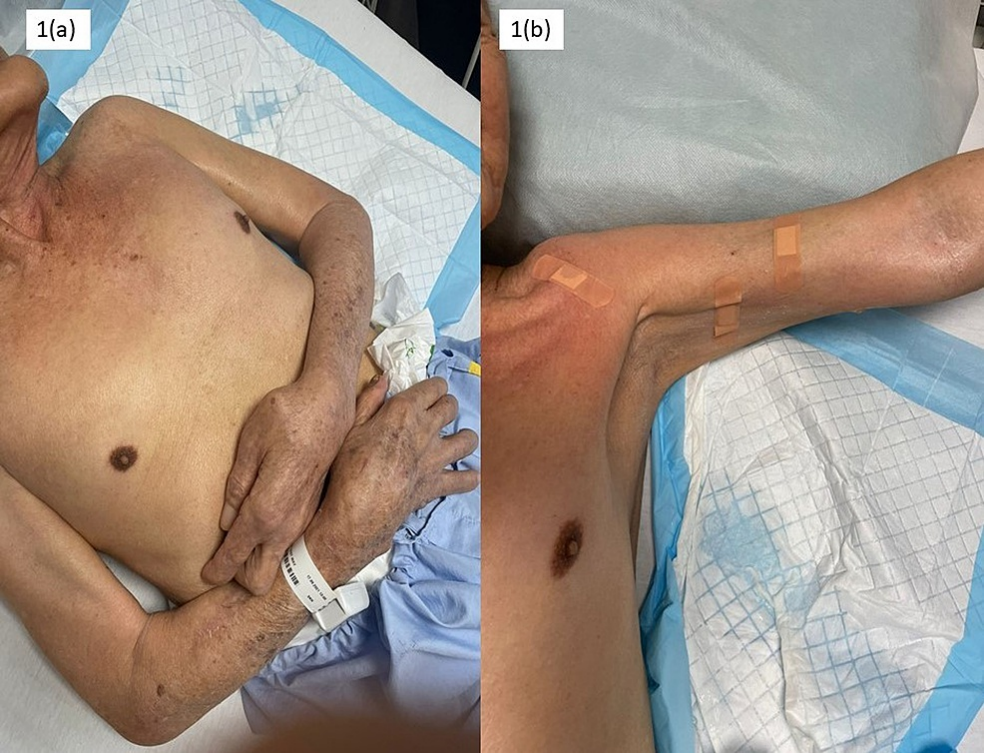
Clinical photos illustrating left upper extremity posture pre and post-alcohol neurolysis and intramuscular nerve blocks. (a) Pre-injection; (b) post-injection.

A 76-year-old gentleman (Mr. S) suffered from bilateral posterior circulation infarcts. He was on outpatient rehabilitation follow-up at our hospital. One year after the stroke, he was assessed to have worsening bilateral upper limb spasticity, pain on passive ranging, and his upper extremities were kept in a flexed and adducted position (Figures 1, Panel a). His caregiver encountered difficulty in upper body dressing and hygiene care.

Physical Findings

The patient was unable to perform any active ROM of the shoulder, elbow, thumb, and finger flexors. On examination, the strength of the upper extremities using manual muscle testing (MMT) was as follows: (a) right shoulder abduction 0/5, right elbow flexion and extension 0/5, right wrist flexion and extension 0/5, and right finger and thumb extension 0/5; and (b) left shoulder abduction 0/5, left elbow flexion and extension 0/5, left wrist flexion and extension 0/5, and left finger and thumb extension 0/5.

Passive shoulder abduction ROM was assessed to be 45° bilaterally. His left elbow extension ROM was assessed to be -90°, left wrist extension ROM was 0°, and left finger and thumb extension ROM was -45°. Moreover, his bilateral shoulder adductor, left elbow flexor tone, left wrist flexors, and thumb and finger flexors demonstrated spasticity on the Modified Tardieu Scale as follows: (a) shoulder adductor: fast passive movement (R1) was 20° and slow passive movement (R2) was 45°; (b) left elbow flexor: R1 was 90° and R2 was 135°; (c) left thumb flexor: R1 was 45° and R2 was 90°; (d) left finger flexors: R1 was 20° and R2 was 45°.

We further assessed the left shoulder using sonographic assessment which did not suggest any ultrasound features of adhesive capsulitis which might have contributed to the shoulder-restricted ROM.

Clinical Management

The patient received a comprehensive rehabilitation program, which included three months of inpatient rehabilitation. The program consisted of intensive daily stretching and strengthening exercises, neuromuscular electrical stimulation, activities of daily living (ADL) training, as well as caregiver training to continue stretching exercises at home. He was assessed to be unsuitable for oral antispasmodic agents such as Baclofen in view of increased daytime drowsiness and somnolence during his inpatient stay and at home. He was also not agreeable for orthotic splints to be applied due to pain and discomfort.

Despite receiving a rehabilitation program, the patient remained symptomatic for his regional spasticity, reporting pain and spasms on performing his ADL. Given the difference in R2-R1 indicating the dynamic component of the spasticity, an informed decision was made to administer alcohol injections to the selected muscles and neurolysis to the nerves involved. Specifically, bilateral pectoralis major intramuscular alcohol blocks and alcohol neurolysis of his left musculocutaneous and left median nerves were performed. Botulinum toxin with alcohol neurolysis was discussed with the patient and his family but was declined due to financial concerns.

Specifics of the Intervention

**Figure 2:**
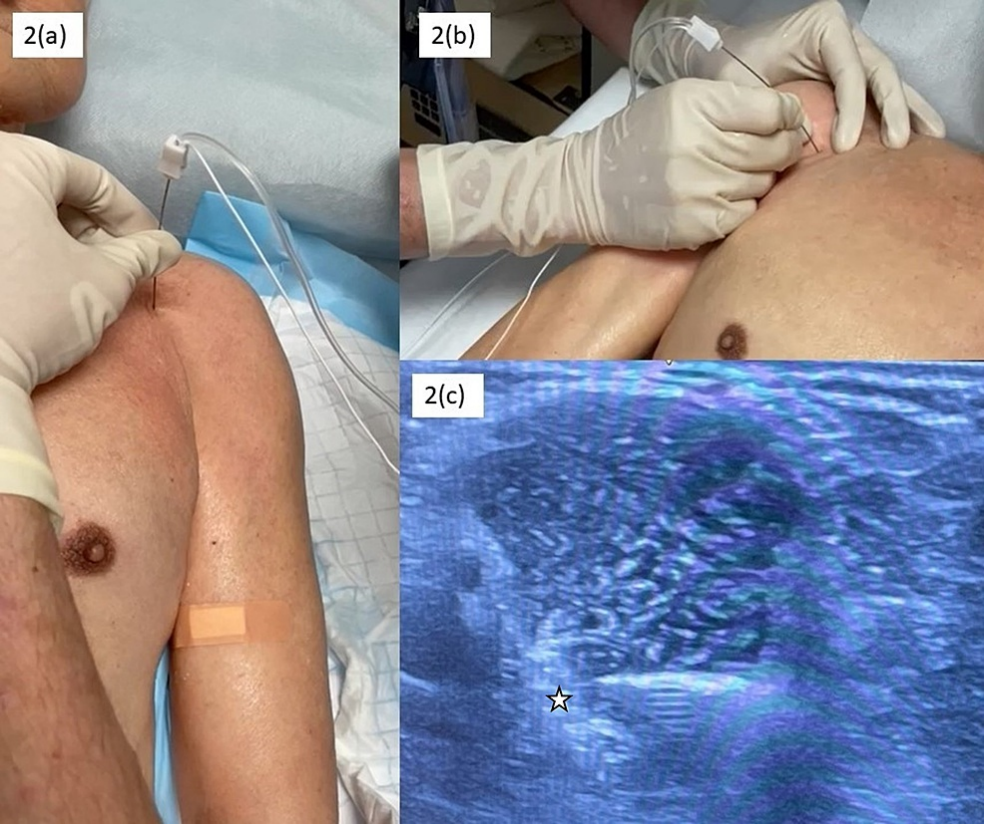
Clinical photos illustrating the procedures. (a) Motor point alcohol injection of the left pectoralis major using nerve stimulator of current between 1 and 2 mA. (b) Motor point alcohol injection of the right pectoralis major. (c) Ultrasound image illustrating alcohol neurolysis of the musculocutaneous nerve. The images show the needle entry via in-plane injection technique advancing toward the star indicating the location of the musculocutaneous nerve.

We administered 75% alcohol (15 mL of 100% dehydrated alcohol with 5 mL of 1% lignocaine). The left musculocutaneous nerve was targeted using ultrasound guidance between the biceps and brachialis with 5 mL of 75% alcohol. Similarly, the median nerve was targeted with ultrasound guidance using an equal amount of alcohol. The initial intention was to further target the lateral pectoral nerve for relieving shoulder adductor spasticity; however, the patient could not tolerate shoulder positioning (shoulder external rotation) to facilitate better visualization of both the lateral pectoral nerves. As such, a decision was made to proceed with 2.5 mL of 75% alcohol/lignocaine in each of the pectoralis major muscles (Figures 2a-2c).

Outcomes

Immediately post-intervention, improvement in the right shoulder adductor and left upper extremity spasticity was noted (Figure 1, Panel b). Six weeks and three months after the intervention, spasticity and joint ROM were improved, indicating the positive therapeutic effect of alcohol on spasticity of the upper extremities. The caregiver reported easier daily nursing care such as upper body dressing, bed to chair transfer, and better hygiene care of the skin of the axilla, elbow, and palm at six weeks and three months. In addition, there was an improvement in bilateral upper limb strength at six weeks and three months with MMT as follows: (a) right shoulder abduction 2/5, right elbow flexion and extension 2/5, right wrist flexion and extension 2/5, and right finger and thumb extension 2/5; and (b) left shoulder abduction 1/5, left elbow flexion and extension 1/5, left wrist flexion and extension 1/5, and left finger and thumb extension 1/5.

Case two

**Figure 3:**
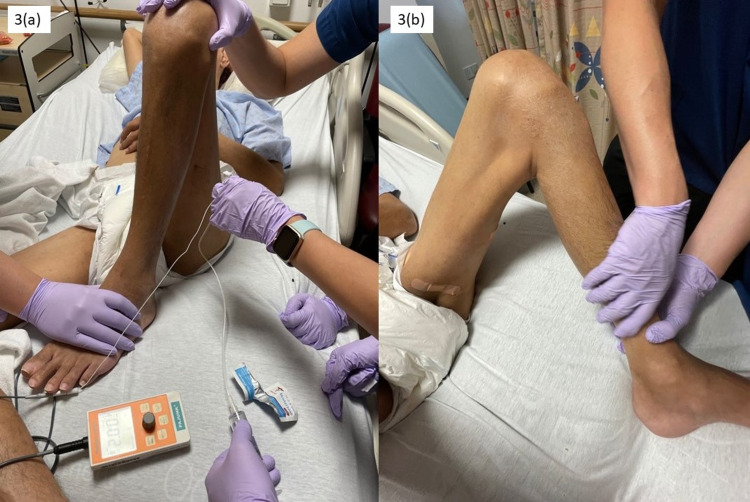
Clinical photos illustrating alcohol motor point injection of the left hamstrings and left knee extension passive ROM pre and post-injection. (a) Pre-injection; (b) post-injection.

A 64-year-old gentleman (Mr. A) suffered from bilateral spastic paraparesis secondary to ossification of the posterior longitudinal ligament and T10-T11 cord indentation and was bed and chair-bound. On an inpatient review four months after the initial diagnosis, he was assessed to have spasticity of his bilateral lower limbs, which kept him in a flexed position (Figure 3, Panel a). There was pain during the transfer from the bed to the wheelchair, and the caregiver encountered challenges in lower body dressing due to flexed knee and skincare of the popliteal fossa.

Physical Findings

No active ROM for the right hip and knee were elicited. The passive ROM was as follows: (a) right hip extension was -80°, and left hip extension was -90°; (b) right knee extension was -140°, and left knee extension was -170°; and (c) bilateral ankle dorsiflexion was 0°.

Spasticity was noted in both the right hip and knee flexors using the Modified Tardieu Scale as follows: (a) right knee flexor: R1 was 30° and R2 was 80°; (b) left knee flexors: R1 was 20° and R2 was 45°.

Clinical Management

The patient received inpatient physiotherapy which included bilateral lower limb stretching and strengthening exercises. He was given a trial of oral spasmodic agents baclofen and tizanidine, which did not result in significant improvement of his spasticity or ROM. He was not suitable for orthotic splints due to severe pain experienced even on slight passive stretching. The patient experienced severe pain from spasticity on performing his ADL and when assisted by the caregiver. He declined botulinum toxin administration to the hamstrings due to the cost involved.

An informed decision was made to perform alcohol neurolysis to the sciatic nerves but the patient declined perineural nerve injection. Upon further discussion, he accepted an alternative strategy of targeting bilateral hamstring major motor points with alcohol.

Specifics of the Intervention

**Figure 4:**
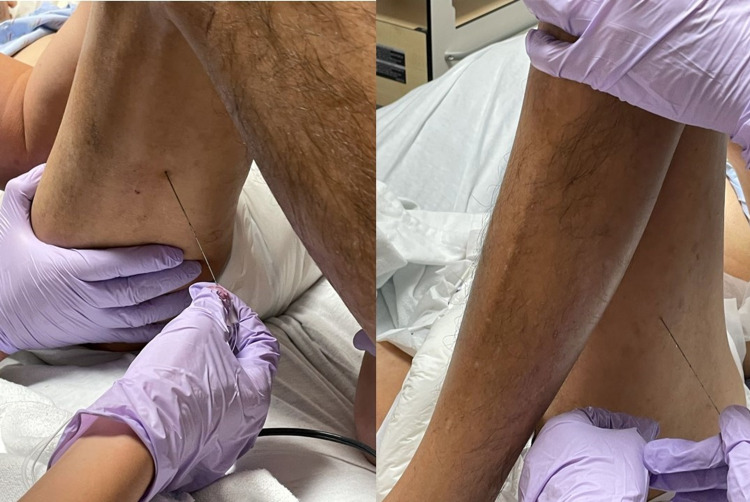
Clinical photos illustrating alcohol motor point injection of bilateral hamstrings. (a) Right hamstring alcohol motor point injection; (b) left hamstring alcohol motor point injection.

We administered 15 mL of 100% dehydrated alcohol diluted with 5 mL of 1% Lignocaine (75% alcohol). The stimuplex needle was inserted into bilateral hamstring motor points using 2 mL of 75% alcohol/lignocaine per motor point (Figures 4a, 4b).

Outcomes

Immediately post-intervention, improvement was noted on the Modified Tardieu Scale, knee ROM, and pain (Figure 3, Panel b). At two weeks, we noted sustained improvement. Table 1 summarizes the outcomes of both patients after the interventions.

**Table 1:** Outcomes of Mr. S and Mr. A after interventions with alcohol. ROM: range of motion; VAS: Visual Analogue Scale

	Spasticity	Joint ROM	Pain	MMT	Adverse events
Mr. S immediate post-treatment	Left shoulder: R1, 45°, R2, 90°. Left elbow flexor: R1, 120°, R2, 160°. Left thumb flexor: R1, 80°, R2, 100°. Left finger flexor: R1, 45°, R2, 80°	Left shoulder abduction: 90°. Left shoulder extension: -20°. Left wrist extension: 30°. Left thumb extension: 10°. Left finger extension: -10°	VAS 3/10	Right shoulder abduction 0/5. Right elbow flexion and extension 0/5. Right wrist flexion and extension 0/5. Right finger and thumb extension 0/5. Left shoulder abduction 0/5. Left elbow flexion and extension 0/5. Left wrist flexion and extension 0/5. Left finger and thumb extension 0/5	No edema, hematoma, or hyperesthesia was noted at all follow-ups
Mr. S at six weeks	Left shoulder: R1, 45°, R2, 90°. Left elbow flexor: R1, 120°, R2, 160°. Left thumb flexor: R1, 80°, R2, 100°. Left finger flexor: R1, 45°, R2, 80°	Left shoulder abduction: 90°. Left shoulder extension: -20°. Left wrist extension: 30°. Left thumb extension: 10°. Left finger extension: -10°	VAS 2/10	Right shoulder abduction 2/5. Right elbow flexion and extension 2/5. Right wrist flexion and extension 2/5. Right] finger and thumb extension 2/5. Left shoulder abduction 1/5. Left elbow flexion and extension 1/5. Left wrist flexion and extension 1/5. Left finger and thumb extension 1/5.	
Mr. S at three months	Left shoulder: R1, 70°, R2, 100°. Left elbow flexor: R1, 120°, R2, 160°. Left thumb flexor: R1, 80°, R2, 100°. Left finger flexor: R1, 60°, R2: 90°	Left shoulder abduction: 100°. Left shoulder extension: -20°. Left wrist extension: 30°. Left thumb extension: 10°. Left finger extension: 0°	VAS 2/10	Right shoulder abduction 2/5. Right elbow flexion and extension 2/5. Right wrist flexion and extension 2/5. Right finger and thumb extension 2/5. Left shoulder abduction 1/5. Left elbow flexion and extension 1/5. Left wrist flexion and extension 1/5. Left finger and thumb extension 1/5	
Mr. A immediate post-treatment	Right knee flexor: R1, 45°, R2, 90°. Left knee flexor: R1, 40°, R2: 80°	Right knee extension: -90°. Left knee extension: -100	VAS 5/10	Bilateral hip flexion/extension 0/5. Bilateral knee flexion/extension 0/5. Bilateral ankle dorsiflexion and plantarflexion 0/5	No hematoma or hyperesthesia was noted at all follow-ups. There was only transient pain experienced during the procedure
Mr. A at two weeks	Right knee flexor: R1, 45°, R2, 90°. Left knee flexor: R1, 40°, R2, 80°	Right knee extension: -90°. Left knee extension: -100°	VAS 5/10	Bilateral hip flexion/extension 0/5. Bilateral knee flexion/extension 0/5. Bilateral ankle dorsiflexion and plantarflexion 0/5	

## Discussion

Motor point block using phenol or alcohol has been described in the treatment of spasticity [3,4]. A summary of selected studies describing alcohol motor blocks is presented in Table 2.

**Table 2:** A summary of studies on the employment of alcohol intramuscular blocks in the treatment of spasticity. VAS: Visual Analogue Scale; MAS: Modified Ashworth Scale; FIM: Functional Independence Measure

Author and year of publication	Study design	Number of patients	Types of intervention described	Percentages of alcohol used	Injection guide	Outcome measures	Adverse effects
Uchikawa et al. 2008 [10]	A cohort study on spinal cord injury patients with neurological level of injury at C5	7	Subscapularis motor block)	0.2–0.3 mL of 5% phenol	Surface landmarks. Patients were positioned on one side, with the arms propped to produce as much winging of the scapula as possible. The needle was inserted under the medial edge of the scapula at the level of the spine of the scapula with electrical stimulation of 1 mA to locate the motor point	Improved shoulder flexion, passive ROM abduction, external rotation, VAS, and eating FIM. No improvement noted in the shoulder MAS scores	Nil
Kong and Chua 2002 [3]	A cohort study of post-stroke patients with finger flexor spasticity	30	Flexor carpi ulnaris, flexor carpi radialis, flexor digitorum superficialis, flexor digitorum profundus, and the flexor pollicis longus	50% ethyl alcohol	Surface localization of motor points using standard electromyogram texts and use of neuromuscular stimulator with a current of 1 mA or less	Improvement in MAS, finger ROM at four weeks, three months, and six months but no improvement in motor control	Dysesthesia lasting for an average of one week

Alcohol or phenol offers several advantages in spasticity management [5], and the rapid onset facilitates serial casting. Alcohol or phenol cost less than botulinum toxins, have therapeutic effects on the sensory fibers to further reduce the spasticity reflex arc, and demonstrate potency on large muscle groups [5].

The concentration of alcohol or phenol used

In both of our cases, we utilized 75% alcohol. A wide range of alcohol concentrations, namely, ethanol (50-100%) and phenol (5-12%), have been reported to be effective in post-stroke spasticity [3,4,6,7]. A higher concentration of alcohol was utilized in these two cases aiming for more permanent blocks due to their poor functional prognosis [3]. Ultimately, the concentration of alcohol used determines the extent of action, and the experience of the clinician is a crucial factor [5]. With higher concentrations of alcohol, a more marked inflammatory response can be noted [1].

For phenol, concentrations of greater than 3% phenol should be used. At these higher concentrations, phenol causes Wallerian degeneration and axonal demyelination leading to muscle denervation [1,8]. Low phenol concentrations (less than 3%) would only achieve a reversible anesthetic effect [1,9].

Choice of the target sites

The combined use of alcohol neurolysis and intramuscular blocks assisted in achieving the therapeutic outcomes. In Mr. S, the pectoris major was easily accessible and targeted using motor point blocks, while musculocutaneous and median neurolysis was more suitable for forearm and finger spasticity. For Mr. A, the aim was to reduce spasticity of knee flexors, and rather than targeting the sciatic nerve, local intramuscular blockade of the hamstring complex offered an easier alternative approach.

Therapeutic outcomes

We observed improved spasticity and targeted joint ROM in both patients. For Mr. S, reduced pain and spasm facilitated better hygiene care of the upper extremity with intramuscular alcohol blocks and neurolysis. Likewise, for Mr. A, intramuscular alcohol blocks alone were able to achieve a better sitting posture. With the immediate therapeutic outcomes observed, physicians can consider the prompt implementation of other modalities such as splinting or serial castings [5]. In both patients, physical therapy, stretching, and positioning after the alcohol procedures were able to achieve the therapeutic goals, and hence, serial castings were not necessary.

Cost consideration

Where sources of funding might not be easily accessible for botulinum toxin, combined alcohol intramuscular blocks and neurolysis is a feasible option.

Adverse events

None of the two patients developed any serious adverse effects at higher concentrations of alcohol (75%), which is consistent with a previous study [1]. None of the two illustrated cases were on oral anticoagulants.

Learning points

Motor point blocks with neurolysis are reasonable modalities to treat spasticity. Clinicians need to be trained in alcohol neurolysis and intramuscular blocks using nerve stimulators and/or sonography guidance. Certain muscles (such as hamstrings and pectoris majors) are easily targeted rather than neurolysis of the nerve. Conversely, elbow and forearm flexors consist of many muscles, and neurolysis should be considered. Second, the cost of large doses of botulinum toxin at each intervention setting can potentially be overcome by adding alcohol as a treatment modality. Botulinum toxins might not be a major concern in regions where comprehensive healthcare funding exists. In countries where funding is a concern, clinicians should appropriately consider alcohol injectates. Third, it might be useful in the subgroup of patients who are non-responders to botulinum toxin. Lastly, clinicians should be aware of adverse effects such as temporary dysesthesia, neuropathic pain, or edema post-intervention.

## Conclusions

We illustrated the effectiveness of using alcohol intramuscular blocks with neurolysis for spasticity management in a select group of patients where choice and cost of botulinum neurotoxin are a concern. Clinicians should explore alcohol intramuscular blocks together with neurolysis as part of the overall spasticity armamentarium when pain and function remain suboptimal despite physical therapy, and/or physical modalities to assist in positioning, and after trials of botulinum neurotoxins. This intervention is safe but clinicians should be mindful of the potential adverse effects.
